# Electromyostimulation-Induced Rhabdomyolysis: A Case Report and Comprehensive Literature Review

**DOI:** 10.7759/cureus.95125

**Published:** 2025-10-22

**Authors:** Ward Mallek, Melek Kechida, Anis Jellad

**Affiliations:** 1 Department of Internal Medicine, Fattouma Bourguiba University Hospital, University of Monastir, Monastir, TUN; 2 Department of Physical Medicine and Rehabilitation, Faculty of Medicine, University of Monastir, Monastir, TUN

**Keywords:** electromyostimulation, exercise-induced rhabdomyolysis, muscle enzyme elevation, musculoskeletal rehabilitation, physical exercise

## Abstract

Whole-body electromyostimulation (WB-EMS) is a training method that activates multiple muscle groups through electrical impulses, offering time-efficient benefits for fitness and rehabilitation. However, improper use, particularly at high intensities, poses risks, including rhabdomyolysis. We report the case of a 36-year-old male who developed WB-EMS-induced rhabdomyolysis, which is a complication that may be induced by a single 25-minute session at high intensity. Laboratory findings revealed significantly elevated creatine kinase (19,534 IU/L) and liver enzymes without renal impairment. The patient was managed with rest and oral hyperhydration, leading to a progressive decline in muscle enzyme levels by the fourth day and total normalization after six days. Users and health professionals should be aware that rhabdomylysis is a complication of WB-EMS and should mitigate risks by monitoring and emphasizing adequate hydration.

## Introduction

Whole-body electromyostimulation (WB-EMS) is a widely used training method that stimulates multiple muscle groups through electrical impulses [1]. It is favored by athletes to enhance performance and reduce injury risks [2] and by sedentary individuals to improve fitness and overall health [3,4]. WB-EMS involves wearing a specialized suit with six to ten pairs of electrodes targeting major muscle groups, making it beneficial for those unable or unwilling to engage in traditional exercise [5]. However, recent studies have raised concerns about the risks of improper WB-EMS application, particularly at high intensities during initial sessions [6,7], which can lead to muscle damage and rhabdomyolysis [7,8]. Here, we report a case of WB-EMS-induced rhabdomyolysis with a literature review of such reported side effects to raise awareness among users about this threatening complication.

## Case presentation

**Figure 1:**
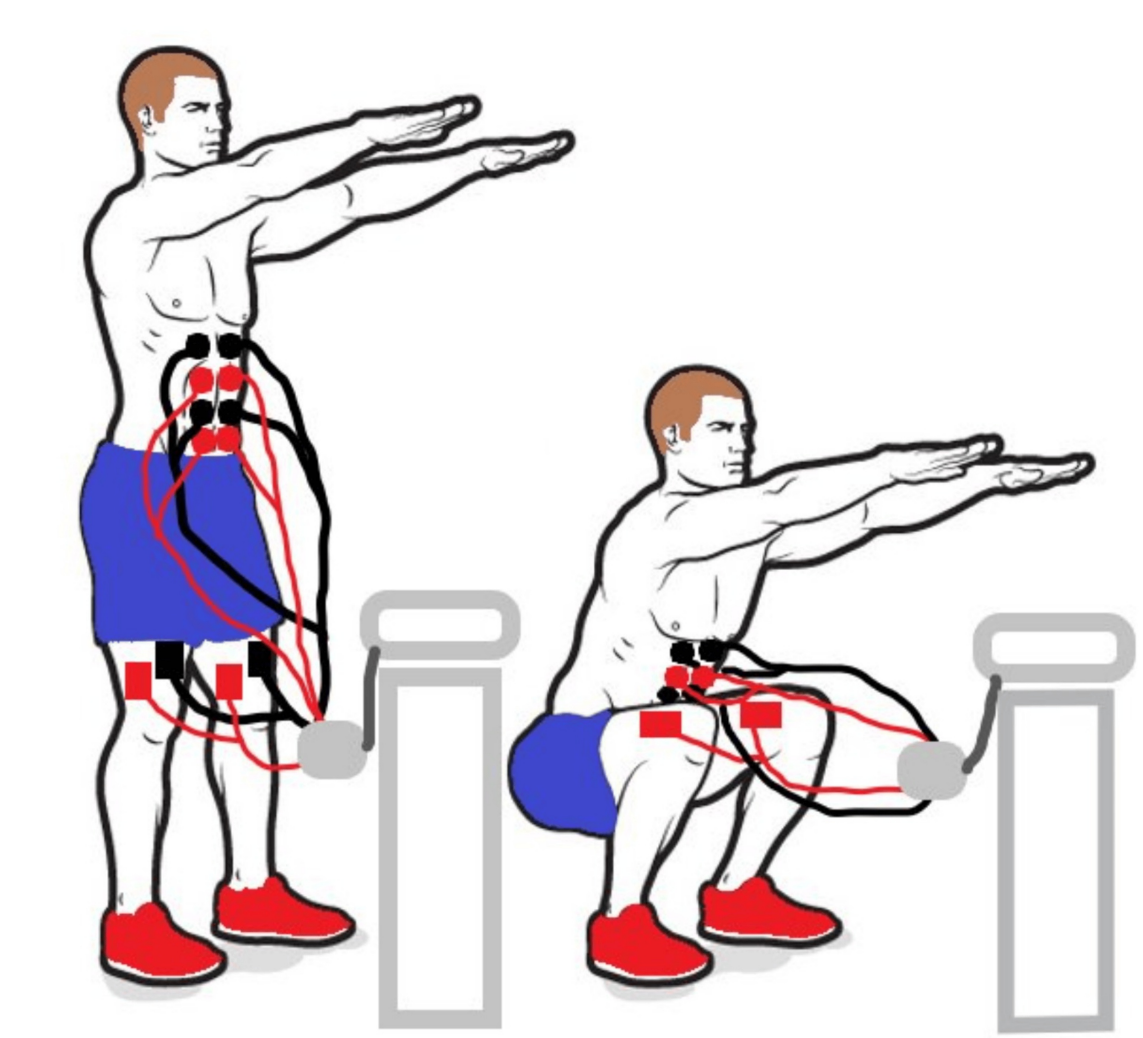
Squat exercise with electromyostimulation (electrodes are placed over the rectus femoris, vastus lateralis, vastus medialis, and rectus abdominis muscles). Image credit: Dr. Anis Jellad.

**Figure 2:**
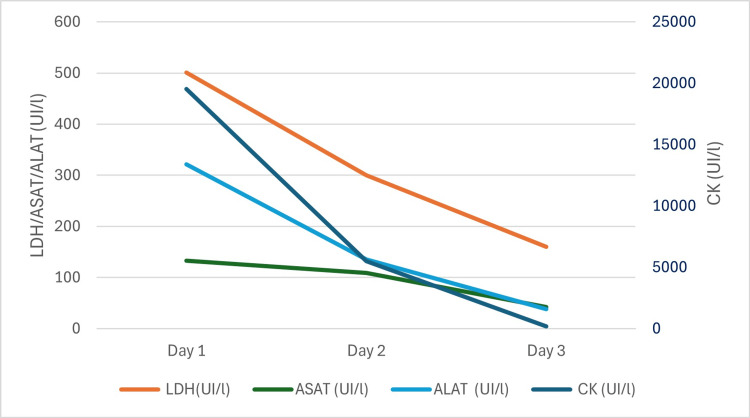
Progressive decline of patient’s muscle enzyme levels over time. LDH = lactate dehydrogenase; ASAT = aspartate aminotransferase; ALAT = alanine aminotransferase; CK = creatine phosphokinase

A 36-year-old male, with no previous medical history, underwent a routine medical workup, which revealed significant elevations in muscle enzymes. He reported undergoing a WB-EMS session 72 hours before the routine workup, at an average intensity level of 70/100 for 25 minutes, equivalent to approximately four hours of conventional exercise. The session targeted multiple muscle groups, including the trunk (abdominal and lumbar muscles), legs (quadriceps, hamstrings, and gluteal muscles), and upper body (biceps brachii, trapezius, latissimus dorsi, and pectoralis major and minor) (Figure 1). The patient had no prior medical or family history of myopathies or medication use and denied recent trauma or substance use. He reported no muscle pain or stiffness and had no muscle weakness on examination. Laboratory tests revealed elevated creatine phosphokinase (CK) (19,534 IU/L), lactate dehydrogenase (LDH) (501 IU/L), alanine aminotransferase (ALT) (133 IU/L), aspartate aminotransferase (AST) (321 IU/L), and normal creatinine (99.8 µmol/L). Thyroid-stimulating hormone was within the normal range, ruling out hypothyroidism. Further workup included inflammatory markers, dot myositis, antinuclear antibodies, viral serologies, electromyography, and imaging showed no abnormalities, ruling out inflammatory myositis or autoimmune diseases. Management consisted of rest and oral hyperhydration (3 L/day), leading to a progressive decline in muscle enzyme levels by the fourth day (CK to 5,506 IU/L, LDH to 300 IU/L, ALT to 109 IU/L, and AST to 135 IU/L) and total normalization after six days (Figure 2).

## Discussion

This case shows a complication of WB-EMS in a healthy male with no prior medical history, incidentally diagnosed during a routine medical workup. WB-EMS enhances muscle strength, endurance, and body composition by activating multiple muscle groups through electrical impulses [1,9-13]. It is particularly valuable in fitness and rehabilitation, offering a time-efficient alternative to conventional training, especially for individuals with limited physical capacity [14,15]. However, WB-EMS use at high intensities or without proper supervision raises concerns about adverse effects, especially rhabdomyolysis. Indeed, WB-EMS involves preferential activation of fast-twitch muscle fibers, which are more susceptible to fatigue and damage, particularly during eccentric contractions induced by EMS [7,9,16,17]. As reported in the literature, this condition results in rhabdomyolysis, generating high levels of CK (Table 1). It may occur in previously untrained subjects, as our patient, but also in highly trained professional soccer players [2,10,11]. Rhabdomyolysis may be associated with dark urine or muscle symptoms such as swelling or pain, but may also be asymptomatic, similar to our case [2,10]. The reported cases occurred mostly after the first session of EMS, similar to our patient (Table 2).

**Table 1:** A summary of EMS-induced rhabdomyolysis cases reported in the literature and the present case. WB-EMS = whole body-electromyostimulation; CK = creatine phosphokinase; LDH = lactate dehydrogenase; ALT = alanine aminotransferase; AST = aspartate aminotransferase

Case	Patient details	Event/Complaint after EMS	Interval between event and first session (days)	Laboratory findings						Intervention	Laboratory findings post-intervention		Recovery (days)	Main outcomes
				CK (IU/L)	Myoglobin (ng/mL)	LDH (IU/L)	ALT (IU/L)	AST (IU/L)	Creatinine (µmol/L)		CK (IU/L)	LDH (IU/L)		
Our case	A 36-year-old male, occupational health screening	-	3	19,534	-	501	133	321	99.8	Conservative management + oral hydration	5,506	300	6	Full recovery without complications
Hong et al. (2016) [9]	A 37-year-old female, weight loss program	Muscle soreness in both arms and arm swelling	3	5,387	264	299	120	118	51	IV/Oral hydration	922	185	4	Discharged uneventfully
Kästner et al. (2015) [10]	A 19-year-old male, soccer player	Dark urine + severe muscle pain in the gluteal and femoral regions	3	240,000	6,764	2,935	-	-	92	ICU admission IV/oral hydration (6–13 L electrolyte solution/6–15 L per day)	62, 880	-	3	Discharged from the ICU
Kästner et al. (2015) [10]	A 17-year-old male, soccer player, routine examination by the national soccer team	Modest muscle ache in the femoral region	4	30,170	-	-	254	896	64.5	Conservative management + reduction of training intensity	135	Normal	10	Full recovery without complications

**Table 2:** A summary of EMS stimulation parameters in the literature and the present case. WB-EMS = whole body-electromyostimulation

Case	Stimulation device	EMS characteristics	Targeted muscle groups (N)	EMS days (d)	EMS sessions/day (N)	Session duration (minutes)	Frequency (Hz)	Duty cycle (s)	Total treatment time (minutes)	Total treatment time/day (minutes)	Intensity
Our case	I-motion device	WB-EMS	10	1	1	25	-	-	25	25	70/100
Hong et al. (2016) [9]	Miha Bodytec	Two sessions with knee push-ups (two sets of 15, day off, one set of 30)	8	2	2	20	85	6 on/4 off	40	20	-
Kästner et al. (2015) [10]	-	WB-EMS followed by two regular training sessions	-	1	1	20	-	-	20	20	-
Kästner et al. (2015) [10]	Not specified	WB-EMS following three sessions of conventional strength training	-	1	1	20	-	-	20	20	-

As high levels of CK may lead to severe complications, including potassium balance abnormalities and acute kidney injury, we raise awareness about such a technique, especially in vulnerable patients, as it was recently revised by the Germain consensus recommendations [17], but also in healthy individuals in special conditions, such as the summer season and dehydration. To mitigate risks, EMS protocols should incorporate gradual intensity progression, monitoring of biomarkers such as CK, and professional supervision. Individuals new to EMS or predisposed to muscle injury require extra caution. Symptoms such as muscle soreness, swelling, or dark urine post-session warrant immediate evaluation for rhabdomyolysis to prevent severe complications [18,19]. Even in asymptomatic individuals, routine laboratory tests for rhabdomyolysis may be recommended during EMS use to monitor and mitigate potential risks associated with prolonged exposure to elevated CK levels.

## Conclusions

As WB-EMS is a truly effective substitute for traditional training, it is increasingly becoming the preferred approach for rehabilitation and fitness. WB-EMS, however, might be more susceptible to rhabdomyolysis, which could result in renal failure. Users and medical professionals should be aware of this issue to reduce risks by monitoring and stressing the importance of drinking enough water. To guarantee the safe and efficient application of such a technology, future research should concentrate on developing safety thresholds, standardized protocols, and long-term impact studies.
